# Existing operational standards for field deployments of rapid response mobile laboratories: a scoping review

**DOI:** 10.3389/fpubh.2024.1455738

**Published:** 2024-11-13

**Authors:** Rand Mushasha, Adela Paez Jimenez, Virginie Dolmazon, Jan Baumann, Andreas Jansen, Oleg Nikolayevich Storozhenko, Charbel El-Bcheraoui

**Affiliations:** ^1^Evidence-Based Public Health, Centre for International Health Protection, Robert Koch Institute, Berlin, Germany; ^2^World Health Organization (WHO) Regional Office for Europe, Copenhagen, Denmark; ^3^World Health Organization (WHO), Geneva, Switzerland; ^4^Information Centre for International Health Protection, Centre for International Health Protection, Robert Koch Institute, Berlin, Germany

**Keywords:** rapid response, mobile laboratory, deployment, operational standards, rapid response mobile laboratory

## Abstract

**Introduction:**

Rapid response mobile laboratories (RRML) play an important role in responding to emergencies such as outbreaks and humanitarian crises, working in coordination with national authorities. This scoping review aims to provide evidence to support the development of minimum operational standards for the deployment of RRMLs across the five key workstreams: operational support and logistics, biosafety and biosecurity, laboratory information management system, quality management systems and interoperability and coordination.

**Methods:**

We searched PubMed, MEDLINE, EMBASE and the grey literature focusing on RRML deployment missions. Study characteristics such as year, country, objectives, methods, and findings were extracted and summarized to identify common themes, gaps, and patterns. The results were presented in a narrative format. We ensured methodological rigor by following the Preferred Reporting Items for Systematic Reviews and Meta-Analyses extension for Scoping Reviews (PRISMA-ScR) guidelines throughout the review process.

**Results:**

Out of 163 full-text studies assessed for eligibility, 46 met the inclusion criteria and were analyzed. Six studies addressed the five RRML workstreams. Operational support and logistics are most commonly addressed during pre-deployment phases with a focus on personnel, transport and cold chain management. The application of biosafety and biosecurity protocols is most addressed during the mission execution phase, particularly in the use of personal protective equipment and the implementation of decontamination and disinfection procedures. The laboratory information management system procedures most frequently reported include sample identification and result dissemination protocols. The quality management system workstream overlaps significantly with the other four workstreams, with a strong emphasis on internal and external quality assurance measures. Coordination and interoperability aspects involve maintaining multiple collaborations, ranging from coordinating with local authorities to establishing international partnerships. Common field challenges included interrupted data transfer in areas characterized by poor connectivity and difficulties caused by extreme weather conditions.

**Discussion:**

This review highlights RRML deployment procedures and addresses some critical challenges concerning their deployment. It suggests the provision of a pre-deployment logistics checklist, the use of a pre-determined standardized dataset for inputs to reduce data entry errors and the application of standardized internal and external quality assurance measures.

## 1 Introduction

Infectious disease outbreaks often occur in rural areas or border regions far from national laboratories. An effective response requires a rapidly deployable mobile laboratory to bring the diagnosis closer to the outbreak site and shorten the time to delivery of results (1). Mobile laboratories are composed of individual diagnostic modules which include equipment, procedures, consumables and expertise customized according to the goals of the mission (2). The uses and contents of mobile laboratories have evolved greatly throughout the years, starting from a simple sample collection kit and light microscope during a melioidosis outbreak in 1997 to fully self-reliant vehicles containing the most recent molecular diagnostic and biocontainment tools used in the West Africa Ebola virus outbreak in 2014–16 (3). Mobile laboratory capacities are a core asset for responding to emergencies, in coordination with national authorities, coordination bodies [e.g., Global Outbreak Alert and Response Network (GOARN) of the World Health Organization (WHO)] and other operational assets (e.g., Emergency Medical Teams, Rapid Response Teams) (4). Between 2014 and 2022, mobile laboratories were deployed to support the response to health emergencies and humanitarian crises in Eurasian and African countries and have therefore provided diagnostic support in hard-to-reach areas during outbreaks such as Ebola and covering peak demands over the COVID-19 pandemic. They have also provided support to Member States experiencing humanitarian crises in the Middle East and eastern Europe, and in pandemic preparedness in Central Asia by providing on the spot training to increase national capacities (5). Deployable mobile laboratories are an essential part of the global health emergency workforce that provides surge capacity aimed at strengthening the preparedness and response of Member States. Rapid Response Mobile Laboratories (RRMLs) are distinguished from mobile laboratories that are used for routine support of national public health systems in the sense that they can be mostly used in times of emergency while supporting public health capacities.

The RRML deployment cycle covers the various stages of operational, tactical and strategic decision-making throughout deployment and can be divided to five main phases, from the initial request for support to the end of the mission and return or transition for subsequent missions (6). The five phases are as follows:

Phase 1 – Mission Assignment:

Initiation of a country’s request for assistance, assessment of mission feasibility, and dialogue between stakeholders to determine mission parameters and objectives.

Phase 2 – Mission Specification:

Tailoring the RRML response based on gathered information, emphasizing a modular approach to ensure scalability and adaptability, and identifying key mission criteria.

Phase 3 – Mission Execution:

Deployment and setup of the RRML at the designated site, communication and coordination with national and international stakeholders, and continuous reassessment of support needs.

Phase 4 – End of Mission:

Reassessment of mission parameters and local situation, agreement on next steps such as continued deployment or repatriation, and completion of necessary activities including restoration and equipment transfer.

Phase 5 – Intermission: Post-mission debrief and evaluation in order to identify necessary modifications or adaptations to the RRML structure, equipment and procedures.

Throughout the years, a series of experts’ consultations were conducted by the WHO Regional Office for the guidance for RRML classification. This was published in 2021 by the WHO Regional Office for Europe and provides comprehensive details on the three-layered classification system (7). The RRML classification system identifies five types of RRML according to their scale of capabilities as follows:

Type I, highly compact: highly mobile, compact laboratory units; equipment can be expanded and composed of 1–3 individual units.

Type II, box based: box-based mobile laboratory units; equipment can be expanded and composed out of more than 3 individual units.

Type III, medium scale: self-contained laboratories in mobile vehicles that are generally single units.

Type IV, large scale: self-contained large mobile laboratories depending on mission needs and desired capacities.

Type V, full scale: self-contained laboratories for stationary or mobile diagnostics that can be expanded and composed of more than one laboratory.

The classification aimed to define laboratory structure and to provide a foundation for the development of RRML minimum operational standards (MOS) (7) to streamline establishment and operationalization of mobile laboratories in field settings. Global standardization will contribute to:

- assuring quality and safety of mobile laboratories operations
- increasing interoperability of mobile laboratories with other operational assets in emergencies.

WHO mobilized experts, including from GOARN partner institutions (2) to develop MOS across five workstreams. The workstreams are, in brief, as follows:

- Operational Support and Logistics (OSL): focusing on the deployment of RRML units and in-field logistics, including custom clearance arrangements, material transport, in-field setup as well as safety and security in the fields.
- Laboratory Information Management System (LIMS) includes data transmission, minimal data sets for RRMLs and the establishment of efficient in-field communication. The LIMS workstream has now been renamed to Knowledge and information management (KIM).
- Biosafety and Biosecurity (B&B): RRMLs apply a risk assessment approach to mitigate biosafety and biosecurity risk according to minimum standards.
- Quality Management Systems (QMS): procedures implemented at every stage of the RRML life cycle allow the provision of required diagnostic support at a consistently high quality to affected communities.
- Interoperability and coordination: Interoperability enables smooth and timely data exchange and enhances compatibility and coordination between the RRML, and all the different entities involved in emergency response efforts. Coordination encompasses resource allocation and sharing (personnel, equipment, expertise), communication strategies and information exchange, joint planning, and operational synchronization to optimize response efficiency and effectiveness.

The development of RRML MOS was additionally supported by multiple simulation exercises to test and review the standards.

This paper serves as an evidence review of the available literature reporting on mobile laboratory deployment missions to answer the questions of whether the RRML procedures are defined and applied for each of the workstreams, what gaps or challenges exist in the procedures, how the RRML procedures reported in literature informed the development of the RRML MOS and if there is available literature to form the basis to establish a RRML monitoring and evaluation system for a potential future recognition. The evidence provided through this scoping review aims to provide an analysis of the existing standards and procedures for RRML field deployments and will further supplement the development and finalization of the MOS.

## 2 Methodology

We have determined that the study design that best fits our research objectives is that of a scoping review. A scoping review is an exploratory and systematic approach that aims to identify and summarize existing evidence in a broad field of study and is particularly well-suited for mapping the key concepts, sources of evidence, and gaps in knowledge within a specific domain (8). The search focused on literature related to the RRML procedures and development of minimum standards for RRMLs, specifically in the areas of Operational support and logistics (OSL), Laboratory Information Management System (LIMS), Biosafety and Biosecurity (B&B), Quality management systems (QMS) and interoperability and coordination across the RRML deployment cycle. The search will attempt to include all aforementioned types of RRMLs (types I-V). In order to address the research questions, the study examined the selected literature to identify the most commonly utilized procedures in the workstreams for laboratory deployment by deploying agencies, while also considering the WHO draft minimum standards they adhere to.

### 2.1 Database search

Initially, we used the PubMed database to extract relevant literature. We conducted the search on June 12, 2023 followed by a search of OVID database in June 18, 2023. The OVID search included OVID journals, Embase and OVID MEDLINE. The search was run again on September 26th, 2024 on both PubMed and OVID. The search was filtered to include publications from the year 2000 till now. The deduplication function on OVID removed multiple duplicates and we carried out manual deduplication of the results yielded.

The following terms were used for both searches and truncation was used to ensure all different variations of the word “lab” were included:

(“RRML” OR “mobile lab*” OR “field lab*” OR “mobile laboratory response” OR “field deployment” OR “deployable lab*”) AND (2000:2024[pdat])

The selection was tailored to include original research articles, book sections, correspondence and commentaries thereby excluding preprints, editorials, letters to editors, interviews, conference abstracts and supplementary files. Papers were removed during the title and abstract screening stage if the title did not address the topic of interest. We then retrieved the full text of the potentially relevant papers and conducted a full text analysis of the studies to assess eligibility using the predefined inclusion and exclusion criteria summarized below. For more details on the inclusion and exclusion criteria for each of the research questions, refer to Annex 1.

The inclusion criteria cover studies focused on deployment procedures and protocols of mobile laboratories, specifically those addressing one or more of the laboratory workstreams (Quality Management Systems Laboratory Information Management System, Biosafety and Biosecurity, Operations Support and Logistics and Interoperability and Coordination). These include analyses highlighting gaps in RRML procedures and protocols as well as studies discussing challenges encountered during RRML deployment missions. Conversely, the exclusion criteria involve studies unrelated to mobile laboratories, those not reporting on RRML procedures or protocols, and those lacking analysis or discussion of gaps within RRML procedures and protocols.

### 2.2 Synthesis of results

Our data extraction process involved the use of a tabular form in Microsoft Excel 365® for Windows. Key study details, such as author, publication year, deployment phase, study type, deployment objective, workstream coverage, RRML type classification, and deploying agents, were systematically organized. The WHO Guidance for RRML classification (7) was used, where possible, to categorize the RRML types mentioned in the studies. Each selected paper underwent a thorough analysis, with procedures implemented by deploying agents being extracted and classified by workstream. We used the WHO’s RRML MOS draft document to determine the workstream and standard under which each procedure falls. We also followed the structured decision-making approach by *Olga Vybornova and Jean-Luc Gala* (6) to determine which deployment phase each procedure was performed in. The challenges encountered and reported on in the literature throughout the deployment cycle were included in our analysis. The data extraction form in Annex 2 includes study details, procedures implemented and the critical appraisal scores for each of the selected studies. Challenges encountered can be found in Annex 1. To manage the included studies efficiently, we utilized EndNote software, which facilitated sorting, arrangement, and citation of the selected studies. Due to the descriptive nature of the selected studies, we did not perform statistical pooling of all included results but rather provide a narrative synthesis of the results, aggregated by workstream.

### 2.3 Critical appraisal

Given the diverse range of study types included in our selection, a single, pre-existing critical appraisal tool was deemed insufficient. Consequently, we developed a dedicated critical appraisal tool to assess how relevant each of the studies were in answering the research questions. The tool evaluates the outlining of the RRML profile in each study, the study content and approach and whether challenges and recommendations were reflected on. The critical appraisal tool can be found in Annex 1. The evaluation scores derived from this tool indicated varying degrees of relevance among the selected studies.

## 3 Results

### 3.1 Study selection

We also followed the Preferred Reporting Items for Systematic Reviews and Meta-Analyses extension for Scoping Reviews (PRISMA-ScR) guidelines throughout the review process. The checklist can be found in Annex 1 (9). The final analysis included selected 46 studies that met our inclusion and exclusion criteria for addressing our research questions.

**Figure 1:**
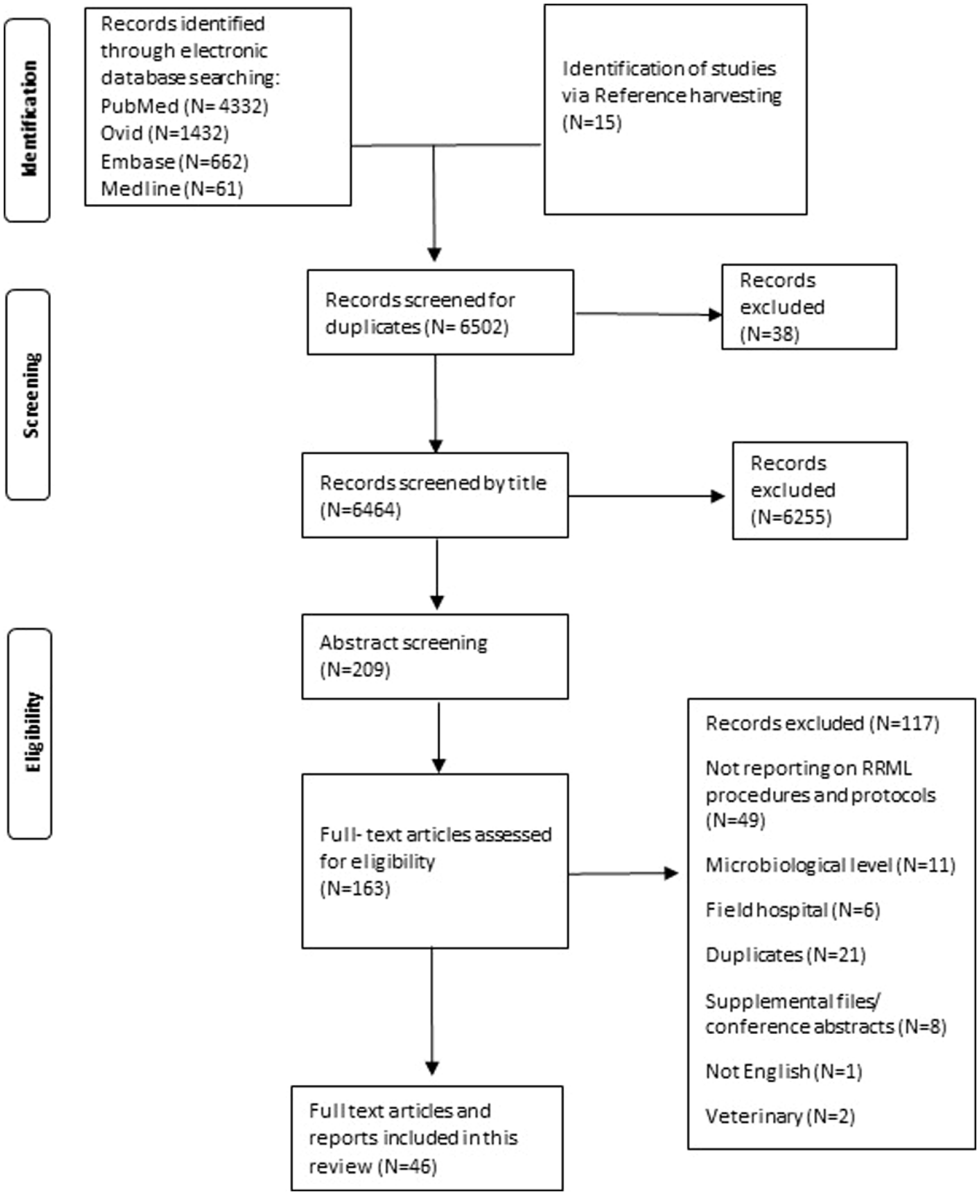


### 3.2 Study characteristics

Some of the key characteristics of the selected literature are as follows:

- Six studies comprehensively addressed all five workstreams (OSL, BB, LIMS, QMS, CIF).
- The workstream with the most extracted protocols and procedures was the biosafety and biosecurity workstream followed by operational support and logistics.
- The primary deployment objective in most cases was a rapid response to infectious disease epidemics, with a focus on Ebola virus followed by SARS-CoV-2 outbreaks.
- The majority of studies adopted a descriptive or observational approach, primarily detailing deployment procedures and strategies while reporting the outcomes of deployment missions.
- Two studies were reporting on field hospital deployments, but included a mobile laboratory aspect and hence were included.
- 17 of the 46 studies addressed deployment challenges encountered either pre-deployment or during deployment, one study focused solely on outlining challenges faced with mobile laboratory use and 11 of them provided recommendations for improvement.
- 17 studies mentioned aspects related to coordination and interoperability. Among these, 13 papers touched upon coordination and three papers on interoperability. One paper briefly alluded to both.

One of the papers did not report on deployment procedures but was a situational analysis analyzing user perspectives, we included it as it provides useful input for our review.

Below are the key findings for each of the five workstreams in the different deployment phases. A tabular more detailed representation of the findings can be found in Annex 2. It is prudent to point out that there is an overlap between various standards in the five different workstreams.

### 3.3 Results by workstream, aggregated by deployment phase

#### 3.3.1 Operational support and logistics (OSL)

Operational support and logistics are most commonly addressed during pre-deployment phases such as mission assignment and mission specification. Based on the reviewed literature, the mission specification phase stood out as the most extensively covered phase, with a focus on personnel, transport, cold chain management, security measures, and staff health and safety protocols. The selected studies lacked, in comparison to the MOS, reporting on laboratory repatriation procedures that would facilitate timely field decommissioning.

##### 3.3.1.1 Mission assignment

During mission assignment, when covering the RRML profile, nine studies emphasized the critical importance of strategic location determination. Key factors for location prioritization included minimizing sample transport time and unobstructed sample transport routes (10–12), having sufficient space (13) with a constant power supply (12), and proximity to treatment units (13–15) and target population (16). A study focused on location determination (17) recommends the location evaluation criteria to include coverage, minimum access, mobility/transportation cost, proximity of service, number of opportunities, and geographical segregation. One study reported on employing regional distributors to facilitate sustainable procurement in East Africa prior to deployment (18). Another study recommends that type II mobile laboratories be set up at peripheral health centers at the outbreak site to shorten diagnostic turnaround time (19).

##### 3.3.1.2 Mission specification

Also, within the RRML profile were protocols concerning personnel, transport mechanisms and equipment needs determination. Sixteen studies focused on building multidisciplinary teams, commonly including 4–6 personnel, though the range varies greatly. Team configurations included different compositions, some including only laboratory and organizational staff (20), and others including team members with specific roles like scientists, technicians (21) and safety officers (22). Some teams operated with rotating personnel (23, 24), while others emphasized the presence of specialized roles such as infection control practitioners and logistics officers (25). Furthermore, some studies highlighted the importance of training prior to deployment (18), having specialized personnel with valid licenses (12) and specific team structures involving buddy systems (26). Additionally, the recruitment of local staff for support was reported (15). Eleven studies included transport, with various modes such as trains, ships, and planes used for transport. Transportation in international deployments via air was the most commonly used method for the shipment of laboratory equipment, materials, and personnel (15, 27–29). Some studies focused on transport setups such as the use of Land cruiser vehicles (18), trucks (16, 30) and Gulf Stream IV jets (27). Others emphasized the collaboration with local and international staff for multi-modal transportation (15) and careful handling to prevent equipment damage (12). Additionally, equipment like vacuum insulated boxes for temperature-sensitive reagents (24) were highlighted. Four studies related to pre-deployment equipment needs’ determination, emphasized equipment selection based on maintenance needs and environmental resistance (18) and conducting continuous needs assessments for essential equipment (27). Essential equipment needs such as air conditioners, thermohygrostats, power input terminals and thermal cyclers were mentioned (12). Implementing a fallback strategy in case of equipment failure included duplicating key components of this equipment (24). Concerning the logistics cold chain plan, six studies highlighted the commitment to preserving the integrity of temperature-sensitive materials during transport and storage by maintaining the required temperature ranges. This was mainly carried out through the use of specialized refrigerators and freezers powered by various means (10, 15, 18, 21, 31) as well as transport with wet ice brick packaging and use of dry ice (28). Effective customs clearance procedures were also noted (24). Security measures were covered in six studies, and these included the establishment of secure zones (14), controlled access (32), strict movement protocols with military escorts and guards (15), physical fortifications such as burglar bars (33), round-the-clock security services (26), and enhanced facility locking mechanisms (31). Staff health and safety measures included safety protocols such as closed-circuit television (CCTV) and alarm system installations, fire escape provisions (34) and recommendations for using non-combustible insulating materials (12). Ensuring sufficient workspace, controlling access, preventing animal and insect entry, and addressing staff well-being through counselling on mosquito protection, dehydration prevention, sun protection, and emotional support were also outlined (27).

##### 3.3.1.3 Mission execution

During mission execution, multiple logistical procedures were repeatedly mentioned in the literature. Fourteen studies covered power supply procedures, where power supply interruption was one of the most common challenges faced during deployment missions. Some of the reported power supply sources were generators with backup uninterrupted power supply (UPS) systems (18), diesel generators, lithium-ion batteries with onboard generators (21) and car batteries with inverters (35). Backup generators, particularly diesel generators, were a primary focus (14, 25, 29, 31, 33). Three water supply procedure implementations were identified. These included the use of water storage tanks, provision of essential equipment for water availability and sewer connections, and safety measures like safety showers and automatic sensor faucets (29). Effective wastewater management was also emphasized, employing methods such as sewage tank sterilization (31) and backwash protection (36). Furthermore, eight sample transport methods were identified, adhering to different guidelines from WHO for packaging and transportation and outbreak response protocols (10, 15, 16, 37). These methods included dedicated vehicles (32), motorcycle couriers (22, 26, 29) and shipping large batches via chartered cargo flights (15). Storage protocols included the use of Biosafety Level 3 (BSL3) gloveboxes for storing reagents, samples and RNA extracts (38) and storage of assay kits in air-conditioned units (26). Freezers were used at temperatures of −20 Celsius for short-term storage of specimens (29, 31) and − 80 Celsius for long-term storage of specimens.

Three security measures were identified, including controlled access to laboratory facilities using card readers (32) and stationed security guards (26). In some cases, military vehicles and armed guards were recruited for laboratory protection along with the implementation of a curfew in high-risk areas (15).

##### 3.3.1.4 End of mission

A study reported on the facilitation of capacity building after mission completion to efficiently transfer Ebola response tools and knowledge to local staff (15). In another study, after one mission and the beginning of another (intermission), relocation and setup of containers has been reported to take place within 1 day with an additional needed day for installation of laboratory furniture, electronic components and security systems, while at end of mission deregistration of the BSL-2 laboratory was filed with the responsible trade supervisory office and health department and a 30-day period had to be complied with. Remaining technical and laboratory equipment was transferred to other facilities for further research and educational projects after a 3-week cooldown (16).

#### 3.3.2 Biosafety and biosecurity (BB)

Applying biosafety and biosecurity protocols can be most seen through the mission execution phase, especially regarding Personal Protective Equipment (PPE) use where a wide range of PPE protocols were applied depending on the protection level needed. Also frequently covered were the various decontamination and disinfection procedures implemented, most often, chemical decontamination was carried out using chlorine bleach. Mostly lacking was the conduction of evidence-based bio-risk assessments for RRML activities.

##### 3.3.2.1 Mission specification

During mission specification, seven studies reported on laboratory management aspects. These included training, the inclusion of a comprehensive review of standard operating procedures (SOPs) for biosafety, biosecurity and laboratory procedures (20). In terms of training, protocols such as ensuring that training curricula covered various aspects of biosafety for specific pathogens like SARS-CoV-2 (38), International Air Transport Association certification (18), proper use of PPE, disinfection protocols (32), laboratory management, risk assessment, and staff protection (25) were included. Another study reported on running a week-long training to cover technical knowledge and practical skills about Ebola assays, with a refresher training if needed and technical knowledge and practical skills (26).

In facility design and infrastructure, specifically containment procedures, the use of biological cabinets and gloveboxes was carried out. Six of the eleven studies reporting on use of biological safety cabinets used class II cabinets for sample preparation and inactivation (12, 29, 32, 34, 39, 40). The use of biosafety cabinets II or III depends on pathogen type and the required level of isolation. In regards to the significance of laboratory design and infrastructure, protocols included the use of easily maintainable and cleanable surfaces (34), having specialized spaces for PPE donning and doffing (36), sectioned indoor layouts with separate zones for various laboratory tasks with filters for the extraction areas (12), and the incorporation of a reagent preparation area with a biosafety cabinet and temperature monitoring equipment (25).

##### 3.3.2.2 Mission execution

During mission execution, facility infrastructure and protective measures included the use of PPE, airflow control and ventilation procedures. Three studies covered air flow and air filtration, and these include integrated closed biosafety isolators with high-efficiency particulate absorbing (HEPA) filters along with negative pressure cascades (21) and UV radiation sources (33), and specifically designed laboratory airflow systems. Ventilation procedures included the use of 100% fresh, filtered air (25), a cascade of low pressure steps to maintain directional airflow, and high air renewal rates, with some facilities achieving up to 25 air changes per hour.

PPE use was one of the most reviewed aspects in the biosafety and biosecurity workstream and was reported on in 16 studies. Protocols included, but were not limited to, wearing appropriate garments such as gowns (12, 22, 25), masks (N95/FFP2/FFP3), eye protection such as goggles (12, 23–25, 32, 37, 39, 41) or face shields (13, 14, 25, 32, 37), and dedicated shoes or shoe covers (12, 22, 25, 31–33). N95 masks were the most commonly used mask type, as reported in six studies (12, 25, 31, 33, 34, 37). The combination and extent of PPE varies depending on the infectious agent being handled and the task being carried out. Decontamination and disinfection procedures were the second most covered biosafety and biosecurity protocols in the mission execution phase. Decontamination protocols typically involve the use of chemicals such as, most commonly chlorine (30, 31, 33, 35), sodium hypochlorite (26, 29, 37, 42) or hydrogen peroxide (21, 25) and also encompass various aspects such as equipment decontamination, surface cleaning, and specimen handling. The frequency and methods of decontamination vary across studies but are consistently emphasized as essential practices to ensure both the safety of laboratory personnel and the integrity of the research environment.

Disinfection protocols such as hand-washing or disinfection stations near laboratory exits are essential, and in cases where these are unavailable, a double glove policy should be in place (34). Various protocols for sample inactivation in high-containment laboratories have been employed. First, studies reported on mobile gloveboxes providing the capacity to contain and inactivate BSL 3 and BSL 4 (18), with some generating negative pressure of 20–40 Pa through a vacuum pressure pump and some were reported to be useful for inactivation of low volume samples (14). Also, a combination of physical and chemical inactivation methods, including water bath incubation and buffer AVL addition were reported to enhance inactivation efficiency during sample handling within biosafety cabinets (31).

Waste management protocols such as hermetically sealing and externally decontaminating nearly full waste bins before transfer to biohazard containers are recommended (20). One study reports that even though a mobile autoclave could be ideal, the safe transfer of solid biohazardous materials to offsite autoclave facilities or onsite incineration, depending on local regulations, should be considered (34). In some cases, waste removal was entrusted to specialized waste contractors (36) and in some cases, waste materials were reported to have undergone chemical inactivation with a chlorine-containing disinfectant, followed by sterilization in a double-leaf autoclave (31). One study reported on regular decontamination and clearance of waste to adhere to BSL-2 containment levels (16).

Various sample handling and transport protocols during mission execution were reported on, such as disinfection and proper sealing of blood collection tubes and vials for secure transport (14), labelling and visual inspection of tubes, and transportation in refrigerated boxes (43). Another study on a mobile laboratory type 2 in a bus deployed for COVID-19 diagnosis reported on storing samples in refrigerators and storing residual saliva samples in −80 Celsius until evaluation (44). Disinfection of sample transport containers surfaces using disinfectant detergents, often chlorine, was reported on in multiple studies. One study reported on samples being unpacked within isolators (21), and another reported on specimens being processed in designated rooms separated from other laboratory areas (33) thereby ensuring a controlled environment for safe handling. The studies reported on the use of suitable PPE while conducting inactivation procedures (29, 31). It is recommended for clinical specimens to be transported following stringent biosafety protocols, this includes adhering to WHO laboratory biosafety manual (45). The protocols mostly included the use of multiple layers of packaging or storage (10, 15, 26, 32, 37) along with a cooling source such as cold packs (10), a cool box (32) or frozen ice packs (15).

##### 3.3.2.3 End of Mission

In nine studies, waste disposal protocols after mission completion were outlined. These include, but are not limited to: labelling of disposable materials and reagents as infectious waste, followed by their incineration (22, 42). Others opted for the transfer of biohazardous waste to the main laboratory for routine disposal processes or removal by host sites if possible (40). Methods for waste disposal varied, with some reporting on utilizing on-site incinerators for solid waste and septic tank disposal for liquid waste (22). Alternatively, the use of mobile autoclaves or offsite facilities for solid waste treatment, along with decontamination and sewage system disposal for liquid waste, was advised (34). Various studies recommended incineration, which was found to be the most commonly used waste disposal procedure, either using oil drums with diesel fuel (33), medical incinerators with high temperatures (29), or onsite incinerators at the Ebola treatment unit (26). Decontamination and transportation of waste to designated disposal areas, followed by autoclaving or disinfection, were also common practices (13, 25).

#### 3.3.3 Laboratory information management system (LIMS)

In the selected studies, LIMS procedures most reported on included sample identification procedures and result dissemination protocols. Data standardization and implementing a defined dataset for patient and sample records were of importance.

##### 3.3.3.1 Mission specification

One paper (39) reports on taking samples and recording them for traceability based on a unique barcode identifier via a Laboratory Information Management Systems (LIMS) system that is connected to the hospital unit for result transfer. Data protection measures accounted for data storage in unexpected events such as fires, natural disasters and software/hardware failures. Further data security measures such as encryption and data integrity verification are recommended, especially if wireless transmission is to be used. Three studies outlined the implementation of pre-deployment trainings. One study by Affara et al. elaborated on the need to train local laboratory operators in mobile laboratories operations, biosafety and diagnostics. Additionally, the project includes training activities related to emergency responses, such as those for Ebola virus disease outbreaks (18). Other studies mentioned training all staff on the use of data management software to guarantee accurate recording of results (36).

##### 3.3.3.2 Mission execution

One study emphasizes the importance for a laboratory data management system to be adaptable, offering mechanisms for quick adjustments, real-time change reflections, and the ability to delete and modify data without system-wide alterations. They also stress the importance of daily database archiving for data recovery purposes (46).

Regarding data standardization, one paper (15) highlighted the use of a unique data template shared by all laboratories, which is shared monthly with the coordinator of field laboratories. Another study incorporated data standardization into the data loading process along with manually reviewing entries that do not match the expected list (46). This practice, along with the use of standardized spreadsheets with dropdown menus and locked formatting contributed to the reduction of data entry errors. Further, there is a need for data-loading packages that can handle varying data quality levels to account for the unpredictability of outbreaks. This need often leads to the use of Microsoft Excel due to its greater flexibility and reduced need for computer programmers. In this study focusing on LIMS (46), it was reported that national database managers are responsible for consolidating data for in-country use and electronical transmission to the WHO. Additionally, the study highlighted the use of data-loading packages to migrate data from spreadsheets with structural and contextual variations into a consolidated database with an emphasis on software compatibility with the network of use.

In terms of inventory management, one study (15) describes the use of *Finale Inventory* software for stock management, including alerting staff about item statuses to anticipate shortages and prioritizing items close to expiration.

The use of Microsoft Excel is repeatedly highlighted in the literature, and was mentioned in six studies. Data management procedures describe the development of a web-based platform enabling the registration of various data types linked to a unique identifier, facilitating real-time updates and the exportation of results as Excel files with graphical representations (47).

Two studies further emphasize the use of Microsoft Excel 2016 for data management (26, 38). Another paper (48) discusses the use of an administrative dashboard and an electronic patient record application for managing admissions, injuries, and laboratory results. Sample identification was addressed in 10 studies. Sample identification methods focused on the assignment of a unique identification number, be it through recording critical patient demographic information (13), specimen time-stamp details with unique identification numbers (13, 16), allocating an outbreak Id for tracking via a designated investigation form (39) or the allocation of unique numerical laboratory identification numbers for specimens and for the maintenance of a comprehensive specimen register (33). Also, sample identification based on the teams collecting the specimens accompanied by a notification form (15) and further sample identification techniques such as barcodes and case id numbers were utilized (12, 29, 36, 43). One paper reported on the use of anonymous numbers for coding samples and subsequently managing the data in Excel and R (21). In another, the process of labelling individual specimen tubes and matching them with their corresponding forms by two technicians was reported (26), this was followed by entry into the ministry of health’s (MOH) database and then to Microsoft Access.

Six studies covered result dissemination protocols implemented, two studies reported on daily result sharing, where one reported on mailing results once a day to the MOH (46) and another opted to have results shared via Excel through the laboratory head and having the results centralized in a database (15), further emphasizing the importance of interoperability and coordination. Other studies reported on communicating results via submitting reports to the WHO and ministry of health (26, 31). In one mission, results were reported to have been mailed electronically to authorized emails using a reporting template supplied by the MOH and WHO while urgent results were communicated directly to attending healthcare providers by telephone. Reporting of results to family or community members through the field laboratory staff was forbidden (33). Another study on a bus deployed for COVID-19 diagnostics reported communicating directly through emailing patients on their registered email addresses (44).

LIMS quality control procedures such as formatting fields to ensure successful importation into the databases and manual reviewing of records were implemented in one mission (46). In another study, training and data management supervision was provided by the WHO with subsequent monitoring site-visits throughout the mission (26). Another approach was the provision of manufacturer instructions for laboratory personnel regarding LIMS use with the mobile laboratory director reviewing those instructions at least once a year (12).

##### 3.3.3.3 End of mission

One study recommended that a final report and test result record sheet in accordance to national laws should be maintained after mission completion. In this case, result sheets had to have a 5-year retention time (12).

#### 3.3.4 Quality management system (QMS)

The QMS workstream is the workstream that overlaps the most with the other workstreams. The protocols most covered were those concerning internal and external quality assurance measures as well as measures for maintaining equipment during mission execution.

##### 3.3.4.1 Mission specification

Several studies underscored the critical role of comprehensive training in managing disease outbreaks. One study reported that the staff involved in testing, especially in specific disease testing such as SARS-CoV-2, were experienced medical technicians or scientists (40), while another emphasized the importance of having task-specific training especially relating to a specific outbreak and PPE training (10). Another study reported on their staff receiving training on various aspects, such as required SOPs, biosafety practices, risk evaluation, and data analysis (25). Furthermore, a study reported on developing and rolling SOPs by training various laboratories across the East African Community on diagnosing arbo- and hemorrhagic fever viruses, as well as an extensive monkeypox diagnostics training (19). An assessment of a training program on risk communication and community engagement, Water, Sanitation, and Hygiene (WASH), Points of Entry (PoE), and infection prevention and control was reported to be a success (49). Notably, a week-long pre-deployment training program was designed to prepare volunteers for real-life outbreak situations, covering biosafety, diagnostics, and emergency procedures (24, 35). One paper identifies implementing a bio-risk management system when working with infectious agents as an essential step prior to deploying a mobile laboratory, this was done by preparing an SOP for all the procedures for the work to be performed in the local language. It was also recommended to include a matrix risk assessment for hazards identified at each step (34). During mission specification, one study reported on having equipment instructions accessible to laboratory personnel and performing equipment maintenance procedures according to manufacturers’ instructions (12). Furthermore, mobile laboratories are recommended to maintain detailed operating procedures, job descriptions, and organizational charts. In one of the missions, a record of training that includes written assessments of the SOPs by the volunteers was used (35). Two studies reported that their laboratories were compliant with the ISO 15189 quality standards (39, 40). The ISO 15189 is an international standard that specifies the quality management system requirements particular to medical laboratories (50).

##### 3.3.4.2 Mission execution

During mission execution, maintaining confidentiality of results was reported to have been overseen by the leader of each rotation (33). In regards to managing incidents, a designated planning department was responsible for developing and issuing a daily action incident plan and any required situation reports (51) and one of the studies reported that both the WHO and Academic Consortium to Combating Ebola in Liberia (ACCEL) were responsible for providing assistance in emerging challenges such as power cuts or equipment errors (26).

Different equipment monitoring protocols vary from conducting regular weekly environmental sampling for decontamination monitoring (40), to regular overpressure decay tests via dedicated pressure inlets (34). Another study outlined evaluating pressure, temperature changes, power supply and equipment location. As suggested in the guideline by Roh et al., these practices were coupled with validating and calibrating new equipment and inspecting key equipment on a quarterly basis (12). On the other hand, another study reported on conducting instrument maintenance every 2 weeks, carrying out quality control monthly on instruments per site, and running calibration cartridges on all instruments every 6 months over a period of 22 months (15).

Sample quality protocols included refusal of specimens without specimen submission forms and confirmable patient history (33), while another reported on having the same personnel, using matching equipment and reagents, run parallel testing using samples from another laboratory (12). Another study reported on retesting 20% of the samples at a central laboratory (38). External quality assurance (EQA) measures were implemented in various ways, one study reported on enabling regular proficiency assessment of the trained personnel working in the mobile laboratory. Another implemented two EQAs using two positive controls and two negative controls for each batch and with inter-laboratory comparison with the nucleic acid testing base (25). The South African Ebola diagnostic field laboratory took part in two EQA runs conducted by the WHO Country Office, the Centers for Disease Control and Prevention and the European Network for Diagnostics of Imported Viral Diseases (33).

Different approaches to internal quality assurance protocols were reported. In one study, independent verification via partner state reference laboratories and expert teams were employed (18). Another means of internal quality assurance reported on was carrying out regular checks on surfaces such as that of the Biosafety Laboratory 3 (BSL-3) space and anteroom (32), via implementation of bench check-lists and on-site supervisory visits (26), or establishing a monthly review program (12). Other studies reported on comprehensive PPE inspection (13, 22).

##### 3.3.4.3 End of mission

After a mission is completed, quality management procedures such as the inspection of the exterior and functionality of nucleic acid extraction and gene amplification equipment (12) were reported. Two studies reported after-mission feedback, including obtaining feedback from the volunteers (35), documentation of team activities, and preparation of after-action reports (51). Additionally, carrying out comprehensive training for local staff in various operational aspects after initial operational experience was reported (33).

#### 3.3.5 Coordination and interoperability

The aspects of coordination and interoperability commonly reported on include maintaining multiple collaborations, coordination with local authorities, and setting up private/public partnerships and maintaining them throughout the mission phases. Also, important and often overlapping with the LIMS workstream is the setting up of a network infrastructure that is compatible with the local network’s infrastructure.

##### 3.3.5.1 Pre-deployment

Short pre-deployment phases are needed when deploying at short notice, for example during disaster responses. In one of the studies, the creation of a mobile screening and diagnostic station was reported on and carried out by integrating a BSL-2 laboratory into a container-based structure using the expertise and equipment of a non-medical university within a short period of time. This modular facility was able to overcome initial supply challenges by in-house production of test kits and disinfectants and expanded shortly to two more sites (16). Good coordination and collaboration with local authorities such as forensic police forces was reported to have allowed for a mixed concept mobile laboratory, where forensic police forces pooled skills with hospital centers to upscale COVID-19 diagnostics by creating a single autonomous mobile solution, deployable in a very short time (39). An institution with no previous experience in putting an emergency medical team into the field was able to do so thanks to systematic coordination with a healthcare organization present at the disaster site.

Fast deployment, accomplished within 12 h by the Rapid Deployment Force of the uniformed US Public Health Service in 2006, reports on the central role of an incident command system led by a team commander. This response was further refined through the incorporation of enhanced coordination mechanisms, a development taken from lessons learned in the aftermath of Hurricane Katrina (51).

##### 3.3.5.2 Mission execution

During the mission, factors such as maintaining multiple collaborations or coordination with local authorities were reported to lead to successful interoperability and coordination. One study reports on successful coordination between a regional intergovernmental organization of six Partner States, the East African Community (EAC), a European laboratory with a long history-the Bernhard Nocht Institute for Tropical Medicine (BNITM)- and a WHO Collaborating Centre for Arbovirus and Hemorrhagic Fever Reference and Research. On a regional level, the main partners were the respective national Ministries of Health (MoH) and their national public health laboratories. This partnership was guided by two regional bodies convening twice a year, namely the Expert Working Group (EWG) and the Regional Steering Committee (RSC). The successful integration of a mobile laboratory network into national outbreak response systems can be attributed to this effective partnership through the distribution of tasks, where the RSC endorsed EWG recommendations and setup and ensured the commitment on the highest political level with compliance to national regulations (18). Interagency coordination and coordination with local authorities are mentioned as a determinant for mission effectiveness in four studies (10, 18, 21, 26). Combined operation of the field laboratory and the national reference laboratory in outbreaks is recommended in another (23). One additional study describes the laboratory head reporting daily to the coordinator of all field laboratories who, in turn, presented the global situation at the general coordination meeting (15). Furthermore, coordination with and training of local staff to operate the laboratory is described as a success factor in two studies (33, 35).

A public/private partnership between the reference laboratory of the State of São Paulo, the most populated in Brazil, and a private company formed a mobile laboratory that offered free timely COVID-19 testing in the hotspots. This mobile laboratory further elaborated on the importance of the collaboration between scientists, health professionals and government bodies by relating the mobile laboratory software system metadata with the diagnostic and sequencing results that were delivered directly to the Federal Healthcare System, thereby providing real-time information of the circulating SARS-CoV-2 variants (43). Setting up and maintaining compatible computer network infrastructures is of extreme importance. In one study, the Israel Defense Force Medical Corps field hospital was deployed to Haiti following the 2010 earthquake with a computer network infrastructure for a digital medical information administration system and an electronic medical record (48). Another study reported on the development of a multifunctional global laboratory database during the 2014 Ebola Outbreak in West Africa by the WHO’s Emerging and Dangerous Pathogens Laboratory Network (EDPLN) by using standardized spreadsheets with dropdown menus and locked formatting to reduce data entry errors and improve data integrity (46).

### 3.4 Challenges encountered during deployment

Listed below are the field-encountered challenges reported in the literature. The challenges are listed in decreasing order of being reported. Overall, the commonly faced challenges included slow or interrupted data transfer in areas with poor internet access, extreme weather difficulties, and the extensive planning required for sustaining remote laboratories in geographically isolated locations. Additionally, issues arising in staff health and safety and managing energy supply disturbances that is sometimes followed by backup generator failure and consequently cold chain disruption were reported. Patient identification issues and cultural objections impacting specimen collection pose further operational challenges.

#### 3.4.1 Pre-deployment

##### 3.4.1.1 Logistical challenges

Pre-deployment logistical challenges included managing the deployment of multiple mobile laboratories, transporting equipment to remote sites with inadequate road infrastructure (10), long-distance transportation difficulties (27), sourcing HEPA filters internationally (26), staff accommodation, transportation, food and clean water access and security personnel (16) and managing staff accommodation (25). Another challenge is obtaining necessary clearances from various Ministries involved in deployment, leading to delays in activating deployment of mobile laboratories (20).

##### 3.4.1.2 Training

Challenges in consideration of personnel’s training, expertise, attitude, and adaptability in hot-zone deployments during pandemics like COVID-19 (38) were reported, as well as the need for staff familiarity with mobile high-containment biological laboratories (MBSLs) for biosafety and biosecurity training and education (52). Furthermore, the need and shortage of trained personnel and specialists that can safely conduct tests was repeatedly addressed in the literature (16, 25).

##### 3.4.1.3 Staff health

Challenges in handling heavy equipment and their safe unloading in remote locations (10) as well as staff immunization considerations (27) were reported concerns.

##### 3.4.1.4 Geographical segregation

Geographic segregation is defined as the uneven distribution of populations based on socioeconomic, racial, or ethnic factors. This can be seen in distances to healthcare facilities and laboratories which were reported to be significantly greater in rural communities in comparison to urban communities. This segregation can lead to disparities in access to healthcare services including testing and treatment for infectious diseases (17).

#### 3.4.2 Mission execution

During mission execution, sample supply chain and quality as well as power supply and technical hurdles were the most frequently encountered challenges.

##### 3.4.2.1 Sample supply chain and quality

Inconsistent supply chains led to the arrival of samples in various containers, often many days after collection and with clotted blood stuck to stopper lids. Also, occasional issues such as dry swab samples requiring rehydration with lysis buffer and broken wooden shafts that leave sharp splintered ends exposed contributed to decreasing sample quality (22). Furthermore, the inconsistent supply chain for sample containers and transportation often contributed to variable sample quality, especially noticeable during early and convalescent disease stages (23). Another challenge was the need to process samples in smaller numbers, which often leads to the prioritization of samples over others (38). Also reported as challenging were the poorly organized specimen delivery systems characterized by delayed and late-night specimen deliveries and the delivery of specimens without patient clinical history (15, 33). Sample transport issues (16) and challenges with specimen backlog were reported as well (26).

##### 3.4.2.2 Power supply and technical challenges

Maintenance of cold chain was found to be challenging due to power loss, especially in preservation of temperature-sensitive materials (10). Furthermore, power failures were reported in various missions (23, 29) leading to need for generator use (22) and at times consequent breakdown of petrol generators and breakdown of PCR instruments due to temperature dysregulation (33). Technical challenges included expired cartridges that could not be used (26), disruptions in refilling laboratory supplies and fuel and lack of a − 80 Celsius freezer inside the laboratory for long-term storage (15).

##### 3.4.2.3 Staff concerns

Ensuring staff availability and preventing fatigue during extended deployments (40), as well as coordinating personnel for split-based operations (10) were found challenging. Consideration of personnel training, expertise, attitude and adaptability in hot-zone deployments is of importance (38) and concerns regarding staff security, (27) discomfort and safety risks due to dysfunctional air conditioning (33) as well as 12-h workdays especially in the initial months of deployment (26) have arisen.

##### 3.4.2.4 Community resistance

Resistance by the host community and cultural objections especially in invasive procedures (23) and lack of trust also lead to safety and security risks for the scientific team and health workers (52). Evacuation of response staff after attacks lead to the interruption of activities and slow resumption after such security incidents (15).

##### 3.4.2.5 Database and patient identification

Identical names and multiple identifiers (22, 23) as well as challenges with laboratory requisition forms (26) lead to difficulties linking patients to their results. Also, result dissemination to the central laboratory was also found to be challenging (26). Ensuring consistent and error-free data during high staff turnover (and use of multiple data collectors), and records being at the specimen level instead of the patient level were among the described challenges. Furthermore, the lack of a unique patient identification system available at the beginning of a deployment mission makes linking data retrospectively to the patient level and to other data sources such as burial, clinical, and surveillance data challenging.

##### 3.4.2.6 Connectivity

Ensuring uninterrupted data transfer or voice communication proved to be challenging because of poor internet access (10, 29, 33, 40).

##### 3.4.2.7 Space and infrastructure

Lack of space inside the laboratory, especially constraints of the glovebox and portable PCR machine (38) as well as lack of onsite storage for “live” blood samples and adapting to the confined space of a mobile laboratory (26) were limitations arising with setting up mobile laboratories in smaller spaces. Also challenging was setting up in remote locations with inadequate infrastructure, including refrigeration and analysis equipment (25).

##### 3.4.2.8 Environmental challenges

Environmental challenges such as high temperatures, high humidity and heavy rainfall may lead to the decomposition of elements of the mobile laboratory (29). Rainfall may also damage the glovebox, rendering it inoperative (26). Extreme weather conditions such as snow, wetness or slippery surfaces may also lead to issues with maneuverability as well as exceeding the vehicles’ climate control capacities (40).

## 4 Discussion

### 4.1 GOARN, COVID-19’s impact and the development of the workstreams

The key functions of GOARN include alert and risk assessment, capacity building and training, rapid response capabilities and operational research (53). The GOARN’s operational support team (OST) is based at the WHO and is responsible for facilitating the day to day running of the network and coordination of outbreak response missions, network activities and communications (53). GOARN’s response to the COVID-19 pandemic highlighted its ability to adapt and deploy experts effectively despite significant operational challenges such as border closures and visa delays. However, it also exposed critical weaknesses, including a reliance on international assistance due to a shortage of local expertise and delays caused by evolving travel restrictions. The pandemic underscored the need for improved focal point engagement, streamlined deployment processes, and better integration of virtual support to enhance future outbreak responses (54). As the pandemic progressed, it became more evident how countries with weaker health systems are more negatively impacted. Strengthening in-country capacity by ensuring better health system preparedness is needed to mitigate the impact, respond to the consequences, and adapt for future public health emergencies (55). Lessons learned from this global health emergency justify the development of MOS across various workstreams (OSL, LIMS, B&B, and QMS). These workstreams represent critical areas where updated standards and procedures can enhance the effectiveness and efficiency of RRMLs in future global health emergencies.

### 4.2 Procedures implemented in each workstream

The scoping review outlined the various procedures implemented in each workstream across different mission phases. OSL were primarily focused on in pre-deployment stages, highlighting the importance of strategic location determination with an emphasis on the reduction of sample transport time and access to facilities. Mission assignment criteria, especially in terms of initial consideration of a laboratory’s request to deploy, are lacking in the literature. Pre-deployment logistics are a cornerstone in the deployment cycle and the possibility of preparing a pre-deployment checklist that is accessible to deploying teams should be considered. Having RRMLs adhere to a set of standards and then applying such a checklist when deploying could set the basis for a future recognition process.

BB procedures were predominantly implemented during mission execution, highlighting the importance of implementation of PPE protocols that vary according to the task performed and level of risk. Furthermore, decontamination measures that maintain the safety of laboratory personnel and the integrity of mobile laboratory environments are an essential aspect of biosafety, especially in remote outbreak locations, and that could to minimize the risk of infection. LIMS procedures were integrated across the different mission phases including aspects such as sample identification and data standardization with a focus on the importance of having a predetermined data set for inputs. Standardizing datasets (when possible) greatly aids the reduction of errors and in reporting results. Result dissemination protocols could lead to issues with data security and adequate data encryption methods should be implemented. LIMS should provide enough flexibility to ensure interoperability with national databases as well as different stakeholders, such as emergency medical teams. QMS protocols exist throughout the deployment phases, mainly including internal and external quality assurance measures, equipment maintenance protocols, and sample quality control procedures, all of which are crucial in ensuring the success of deployment operations. The approaches to implementing quality measures varied greatly across the studies, and a possible mean to standardize quality assurance measures, as well as building a pre- and post- deployment training curriculum for personnel is to be considered.

### 4.3 Minimum operational standards

The analysis of protocols extracted from the literature revealed several potential areas where the MOS for RRMLs may need further refinement. In the selected studies, test turnaround time was often recorded, and served as a measure of how successful the deployment of a mobile laboratory was. We find that the MOS could benefit from the inclusion of test turnaround time as a measure of efficiency. This could provide a valuable tool for assessing the overall effectiveness of RRMLs in responding to infectious disease outbreaks. Many studies reported on setting a specific workflow for their laboratories and we find that the pre-determination of the required workflow inside the mobile laboratory structure can be extremely beneficial in terms of contamination control within the biosafety and biosecurity workstream. As previously mentioned, quality assurance measures varied across the studies. We find that specifying when, and how frequently, a quality assessment should be carried out throughout the deployment cycle under the QMS workstream could be useful for maintaining the integrity of laboratory operations and for a potential subsequent monitoring and evaluation. This can help identify areas in need of improvement, and also provide feedback for future potential deployments. In terms of laboratory type classification, incorporating a laboratory type classification according to the WHO laboratory classification guidance into the MOS could provide clarity in terms of capabilities and expectations for each RRML type, thus aiding in more effective deployment planning and resource allocation. To promote data consistency and efficient reporting, it is advisable to consider building a uniform dataset for patient and sample records within the LIMS workstream. This would facilitate data exchange and interoperability between RRMLs and central laboratories, reducing errors and enabling faster outbreak data reporting. Lastly, a clearer definition of which protocols need to be undertaken under each of the deployment phases, such as a mission specification checklist encompassing the five workstream, could contribute to a more systematic and organized approach to RRML deployments. This could guide deploying agencies in ensuring that essential criteria and procedures are not overlooked.

### 4.4 Overlapping workstreams and standards

The nature of the procedures and protocols outlined in this paper often overlap in more than one workstream. This overlapping nature of the different workstreams emphasizes the importance of a cohesive and integrated approach in setting protocols and standards, and their eventual application in reality. For instance, the effective management of cold-chain logistics was essential not only for OSL but also for BB, emphasizing the importance of maintaining temperature-sensitive materials during transport and storage. Similarly, the integration of LIMS procedures with various aspects of QMS underscores the critical role of data standardization, sample identification, and quality control measures that contribute to enhancing interoperability and coordination and therefore the effectiveness of mobile laboratories during deployments. The significance of the overlap between LIMS and interoperability and coordination is further emphasized through the various studies reporting on setting up a network infrastructure that connects between mobile laboratories and local databases. Generally, QMS is the workstream most overlapping with the other workstreams as quality assurance measures are needed to ensure adequate logistical support, biosafety and biosecurity measures as well as information management.

### 4.5 Recommendations

Overall, and in light of the challenges encountered during the deployment of RRMLs, the need for robust and adaptive MOS could promote preparedness in unexpected situations and improve the standards RRMLs are held at. Here, it is essential to keep in mind available resources in the field, as well as contextual factors surrounding the areas to be deployed to. Despite the best efforts to establish robust MOS and comprehensive protocols, it is essential to recognize that some challenges may continue, or reoccur, due to the unforeseeable circumstances when responding to infectious disease outbreaks or other public health emergencies. In such scenarios, the unexpected nature of events can potentially limit the extent to which certain challenges or recommendations can be preemptively addressed.

Furthermore, one area that requires attention is environmental stresses. These include high temperatures, high humidity and heavy rainfall, among others. Environmental stresses present significant challenges crucial to the deployment and maintenance of RRMLs, as demonstrated in the literature. Studies have shown that these stresses can impact the performance and reliability of the RRMLs. Extreme weather conditions could also affect maneuverability of the laboratories during deployment. The importance of taking environmental stresses into consideration should be emphasized further in the workstreams, specifically during pre-deployment location determination. Also, one aspect of geographical segregation that should be considered is geographically isolated nations such as island nations. Island nations are more severely affected by rising oceans, heavy rainfall and global warming, making them harder to reach. These considerations must be made when conducting a geographical spatial analysis to ensure determining geographically accessible deployment locations on both land and sea.

Below, we present further recommendations by workstream, keeping in mind that some of these would overlap.

#### 4.5.1 OSL

Updating the MOS is of significant importance for enhancing RRMLs’ adaptability and effectiveness. This means that the standards should have flexible supply chain management and consistent cold chain maintenance procedures, have adaptable criteria for equipment and consumables, provide guidelines for remote support, and account for challenges such as power supply reliability and overcoming travel restrictions.

Further, to overcome geographic segregation and improve access, geospatial analysis is essential to identify vulnerable populations and optimize the location of mobile laboratories. As such, accessibility measures can be used to optimize the location to which RRMLs can be deployed. Accessibility measures include how much time and costs are incurred by individuals to reach the mobile laboratories. Furthermore, including a spatial segregation measure relating to defining an inclusive service network (to avoid excluding regions out of potential service areas) is of extreme importance, especially in a pandemic setting where mobility is inhibited.

#### 4.5.2 LIMS

Developing comprehensive standards that enhance data management is of utmost importance. These should include robust data security and privacy measures especially when data is being transmitted internationally, establishing a baseline for LIMS that is functional in low-bandwidth environments and that is compatible with various health information systems to maximize interoperability, defining minimum capabilities for data analysis and reporting to ensure high quality and error reduction, as well as strengthening communication infrastructures and maintenance of secure remote access in the field. Furthermore, a standard on setting up platforms that are able to function in low-bandwidth environments could be of great use. Setting up guidelines for information dissemination to diverse stakeholders is vital for maintaining transparency and fostering collaboration.

#### 4.5.3 B&B

Establishing robust biosecurity measures is important for ensuring the safety of personnel and security of sensitive materials in diverse environments while accounting for challenges that could arise in resource limited settings or extended deployments. It is recommended to include a wide spectrum of pathogens in the standards. Developing guidelines for risk reduction can mitigate risks associated with handling hazardous samples or equipment.

#### 4.5.4 QMS

Adhering to standards can support maintaining a consistent high-quality performance across the RRML deployment cycle. These standards should establish a comprehensive framework that is flexible enough to apply in various contexts, including challenging circumstances. Quality control of diagnostic procedures in challenging environments are vital to maintaining accuracy and reliability of test results. Utilizing previous mission reports and providing comprehensive staff trainings can provide a means for continuous improvement. Enhancing QMS and implementing robust quality assurance measures will address challenges related to equipment handling, sample quality, and overall laboratory functionality, contributing to the effective response in public health emergencies.

### 4.6 Limitations

Our study has a number of limitations. The study primarily relied on published peer-reviewed literature and might have missed unpublished data or grey literature. Additionally, the included studies were limited to a specific timeframe and might not reflect the most recent advancements or changes in the field. As our focus for RRMLs is meant to address their application in public health emergencies, and specifically epidemic control, we recognize the application of mobile laboratories in emergency care, such as stroke ambulances that provide point-of-care diagnostics, or for onboard testing on boats, aircrafts, and helicopters. These diverse applications of mobile laboratories should be considered when developing standards for mobile laboratory deployment. As seen during the COVID-19 pandemic, RRMLs can serve in the response to outbreaks on cruise ships for example (56). Furthermore, the heterogeneity of the reviewed literature and the varying geographical contexts might have influenced the generalizability of the findings. The overlap of protocols across different workstreams may lead to the mis-categorization of certain procedures, potentially resulting in the inclusion of protocols in one workstream that could be more fittingly categorized under another. Also, the potential for missed information during the data extraction process might have resulted in the omission of certain protocols or challenges from the analysis. Lastly, the retrospective nature of the studies analyzed entails that the challenges and recommendations documented in these studies were often formulated after the fact. In the context of public health emergency responses, such as infectious disease outbreaks, the unexpected and rapidly evolving nature of the crisis may limit the ability to foresee and prepare for all challenges in advance. This retrospective aspect implies that certain challenges and recommendations would not be accounted for or actionable prior to deployment.

## 5 Conclusion

RRMLs are valuable tools in health emergencies such as outbreaks and other humanitarian crises, and are able to contribute to increasing both national capacities for response and preparedness. Guiding documents on MOS can help building a future monitoring and evaluation scheme followed by a potential recognition process. This standardization, up to the possible extent, of rapid response mobile laboratories serves to enhance quality of deployment and services provided across the workstream. The selected studies reported on the workstreams (OSL, BB, QMS, LIMS, CIF) in varying degrees, and some emphasis was placed on some protocols in each workstream more than others. The literature provided valuable insight into what protocols are actually implemented during deployment missions and to what extent. The overlap between the different workstreams necessitates the development of relevant yet adaptive and flexible MOS that can be applied to multiple workstreams at the same time. Challenges encountered during deployments are varied and to be expected, as further proven in the literature. Taking these challenges into consideration when developing guidance on MOS is imperative. Mobile laboratories serve an important role in effectively addressing laboratory and diagnostic gaps by providing surge capacities during emergencies, especially in harder to reach areas. As demonstrated by the COVID-19 pandemic and subsequent disease outbreaks, such as the MPox outbreaks in Europe and the Democratic Republic of Congo, the Marburg virus disease outbreak in Rwanda (57), and the cholera outbreak in central and eastern Sudan (58), emerging infectious threats are on the rise, or further detected due to improved surveillance techniques and awareness. This trend, especially in harder to reach areas, emphasizes the need for effective, rapidly deployable mobile laboratories that play an essential role in epidemic control.

## Data Availability

The original contributions presented in the study are included in the article/Supplementary material, further inquiries can be directed to the corresponding author/s.
