# Supplementary material for: Existing operational standards for field deployments of rapid response mobile laboratories: a scoping review
Source: Front Public Health. 2024 Nov 13;12:1455738. doi: 10.3389/fpubh.2024.1455738 (PMC11599251; doi:10.3389/fpubh.2024.1455738)
Supplement: Supplementary file 1 [file Data_Sheet_1.PDF]

Research questions and inclusion and exclusion criteria

1. Are the RRML procedures defined for each work stream and what are the gaps in the procedures?

Inclusion Criteria	Exclusion Criteria
Studies related to the implementation procedures and protocols of mobile lab	Studies not related to mobile labs
Studies that address one or more of the laboratory work streams (QMS, LIMS, BB, OSL)	Studies not reporting on RRML procedures and protocols
Studies analyzing gaps in RRML procedures and protocols	Studies not analysing or discussing gaps in RRML procedures and protocols

2. How have the RRML procedures informed the development of the RRML minimum standards and what are the gaps in the minimum standards across all work streams?

Inclusion Criteria	Exclusion Criteria
Studies examining the relationship between RRML procedures and the development of	Studies not related to RRMLs
Documents analyzing the development and implementation of RRML minimum	Studies not examining the relationship between RRML procedures and minimum standards
Documents discussing the gaps in the RRML minimum standards across the four	Studies not discussing gaps in minimum standards across the workstreams within

3. Is there available literature to form the basis to establish a RRML Monitoring and Evaluation (M&E) system that can be used for future accreditation processes?

Inclusion Criteria	Exclusion Criteria
Studies discussing RRML M&E systems	Studies not related to RRMLs M&E systems
Studies exploring the use of M&E systems in the accreditation* of RRMLs	Studies not discussing the role of M&E systems in a forthcoming accreditation* process
Studies providing evidence for establishing a M&E system for RRMLs	Studies not providing evidence for establishing a M&E system for RRMLs

Critical appraisal tool

1. Classification of RRML type reflecting on* WHO Rapid Response Mobile Laboratory (RRML) classification guidance

Is the mobile lab type stated classified in accordance to the WHO classification guidance?
Yes (1 point)
No (0 points)

*Reflecting on the WHO classification guidance and taking into account its publication date in 2021, it is noteworthy that 10 out of the 43 studies were released subsequent to that year

2. Presentation of procedures by phase of deployment implementation

Does the study present the phase of deployment (mission assignment, mission specification, mission execution, end of mission, intermission) the RRML procedures were implemented in?
Yes (1 point)
No (0 points)

3. Inclusion of the Four Work Streams (QMS, LIMS, BB, OSL)

Does the study include material on the four work streams related to RRMLs: Quality Management Systems (QMS), Laboratory Information Management Systems (LIMS), Biosafety and Biosecurity (BB), and Operational Support and Logistics (OSL)?
QMS included? (1 point)
LIMS included? (1 point)
BB included? (1 point)
OSL included? (1 point)
No work streams included or discussed (0 points)

Study content and approach

4. Evidence-Based Approach

Is the study based on or references existing evidence on RRML deployment procedures and standards, such as existing scientific evidence, standards, guidelines, field reports etc.?
Yes (1 point)
No (0 points)

5. Reporting of at least one outcome measure*

Does the study report on at least one outcome measures related to the effectiveness or impact of RRML procedures or protocols?
Reporting of key outcome measures (1 point)
No reporting of key outcome measures (0 points)

*Key outcome measures such as turnaround time, number of samples taken

Gaps and Recommendations

6. Identification of gaps/ challenges in the deployment process

Does the study identify any gaps in deployment across the deployment phases?
Identification of gaps in the deployment procedures (1 point)
No identification of gaps in the procedures (0 points)

7. Recommendations for improvement

Does the study provide recommendations for improving RRML deployment procedures, protocols, or standards based on the identified gaps?
Recommendations outlined based on identified gaps (1 point)
No recommendations for improvement given (0 points)

Scoring: Total possible score: 10 points

Challenges encountered during deployment

Author	Challenges
**Ballard et al**	• Digital Data Transfer: Slow or interrupted digital result data transfer in areas with poor internet access hindered efficient communication.<br>• E-order Mobilization: Mobilizing a Rapid Response Team (RRT) capable of collecting samples using the DHV COVID test registry was necessary in regions unfamiliar with electronic orders.<br>• Extreme Weather: LabVan operations faced limitations in locations with extreme weather conditions exceeding the vehicle's climate control system's capacity, which is essential for equipment function.<br>• Maneuverability Issues: Certain locations with adverse conditions like snow, wetness, or slippery surfaces posed challenges in maneuvering the LabVan.<br>• Operational Hours: Ensuring staff availability and preventing fatigue during extended deployments in remote and unfamiliar environments posed operational hour limitations.
**Cardile et al**	• Mission Requirement: Managing the deployment of four remote laboratories imposed logistical complexities.<br>• Personnel Split-Operations: Coordinating personnel for split-based operations presented staffing challenges.<br>• Remote Laboratory Setup: Establishing and sustaining remote laboratories in geographically isolated locations required extensive planning.<br>• Equipment Transportation: Transporting equipment to remote sites with inadequate road infrastructure posed logistical hurdles.<br>• Heavy Equipment Handling: Safely unloading heavy equipment at remote locations was a concern.<br>• Equipment Damage: Risk of equipment damage during transportation or malfunction at the site necessitated careful handling.<br>• Communication: Ensuring uninterrupted voice and internet communication for remote laboratories was challenging.<br>• Energy Supply: Generator malfunctions and power loss due to poor fuel quality or wet stacking impacted operational continuity.<br>• Cold-Chain Maintenance: Power loss leading to cold-chain disruption posed challenges in preserving temperature-sensitive materials.
**Flint et al**	• Inconsistent supply chain resulted in samples arriving in various containers.<br>• Samples often received many days after collection, with clotted blood stuck to stopper lids.<br>• The quality of samples remained inconsistent, with no standardized swabbing system in use.<br>• Swab samples were sometimes dry and required rehydration with lysis buffer.<br>• Wooden shafts of swabs were occasionally broken off, leaving sharp, splintered ends exposed.<br>• Difficulty in linking patients with their results due to identical names and multiple identifiers.<br>• Electricity supply challenges at the laboratory, with occasional power failures requiring the use of portable generators
**Frimpong et al**	• Limited space inside the laboratory, especially size constraints of the glovebox and portable PCR machine.<br>• Need to process samples in smaller numbers, requiring prioritization.<br>• Emphasis on safety and security of personnel and equipment.<br>• Consideration of personnel's training, expertise, attitude, and adaptability in hot-zone deployments during pandemics like COVID-19.
**Grolla et al**	• Difficulty in collecting appropriate clinical specimens due to factors such as cultural objections, lack of trained personnel, and inadequate infrastructure.<br>• Resistance in the community to invasive procedures like bleeding and post mortem sampling.<br>• Inconsistent supply chain for sample containers and transportation.<br>• Inconsistent sample quality, particularly in early and convalescent disease stages.<br>• Challenges in linking patients with their results due to multiple identifiers.<br>• Electricity supply challenges at the laboratory, with occasional power failures.
**Levy et al**	• Disorder and lack of control in the medical facility means that resources, which are always limited and insufficient in these situations, will be inadequately allocated and not used to the maximum benefit of the population in need.
**McCunn et al**	• Considerations faced in responding to an international natural disaster, such as long-distance transportation challenges, immunization considerations, and security issues

Author	Challenges
**Paweska et al**	• Poorly organized specimen delivery system such as late-night specimen deliveries and specimens delivered without patient clinical history.<br>• Unsafe packaging causing specimen leakage.<br>• Lack of consistent electricity and water supply leads to reliance on a petrol generator, which also broke down.<br>• Breakdown of PCR instruments due to high ambient temperature and power supply problems.<br>• Dysfunctional air-conditioning units leading to discomfort and safety risks.<br>• Temporary closure of the laboratory due to technical problems.<br>• Inefficient and occasionally dysfunctional internet and 3G cellular network connectivity for communication and result reporting
**Presser et al**	• Technical challenges, including electrical power supply, safe water, sewer connections, laundry facilities, biohazard waste disposal, and internet connectivity.<br>• Generator inefficiency due to oversized generators, resulting in increased fuel expenses.<br>• Environmental challenges in Guinea and Sierra Leone, such as high temperatures, high humidity, and heavy rains lead to rapid decomposition of many elements of the MDLs
**Raftery et al**	• Dealing with a specimen backlog upon opening the laboratory and adapting to the confined space of a mobile laboratory.<br>• Handling a huge workload in the initial months, requiring 12-hour workdays.<br>• Rain damage to the glove box, rendering it inoperative.<br>• Sourcing HEPA filters internationally.<br>• Lack of onsite storage for "live" blood samples, requiring inactivation and overnight storage.<br>• Issues with biosafety during specimen collection and transport, leading to specimen rejection.<br>• Challenges with laboratory requisition forms and patient identifiers, making result matching difficult.<br>• Results dissemination from central to county and facility levels.<br>• Transition plan after a decline in specimen numbers, including instrument and staff redeployment to different facilities<br>• Expired cartridges that could not be used for EVD testing.
**Rao and Bordelon**	• Lack of trust and suspicion from the local population in faraway regions where Pilot MBSLs are deployed. Leading to potential personal safety and security risks for the scientific team and health workers.<br>• The need for staff familiar with MBSLs for biosafety and biosecurity training and education.
**Stanislawski et al**	• Supply and provisioning of consumables and safety equipment during the initial phase of the SARS-CoV-2 pandemic.<br>• Compliance with appropriate BSL and ISO requirements for container-based laboratory setups, specifically adhering to BSL-2 containment level specifications for interior surfaces and waste disposal.<br>• Acquisition of technical equipment during the initial stage of the pandemic.<br>• The need for trained personnel to safely implement PCR and antibody detection tests.<br>• Decentralized sample collection and transport to reduce the demand for biotechnology professionals.
**Guo et al**	• Remote locations with inadequate infrastructure, including refrigeration and analysis equipment.<br>• Shortage of well-trained and experienced specialists, with a need for a complete, independent management and professional group during the SARS-CoV-2 outbreak.<br>• Logistical challenges, including accommodations, transportation for staff, medication, food, clean water, access to electricity, and security personnel.<br>• Lack of a -80°C freezer inside the lab for long-term sample storage, requiring careful packaging and transport to another facility for storage.
**Mukadi et al**	• Interruption of activities after attacks on response teams.<br>• Movement of contacts, suspected, and confirmed case-patients leading to disease spread.<br>• Slow resumption of activities after security incidents.<br>• Evacuation of response staff from outbreak areas during insecurity events.<br>• Delayed implementation of activities.<br>• Disruption in refilling laboratory supplies and fuel.<br>• Delayed sample transportation to the laboratory, particularly for sequencing purposes.

Preferred Reporting Items for Systematic reviews and Meta-Analyses extension for Scoping Reviews (PRISMA-ScR) Checklist

SECTION	ITEM	PRISMA-ScR CHECKLIST ITEM	REPORTED ON PAGE #
**TITLE**			
Title	1	Identify the report as a scoping review.	Pages 1,4
**ABSTRACT**			
Structured summary	2	Provide a structured summary that includes (as applicable): background, objectives, eligibility criteria, sources of evidence, charting methods, results, and conclusions that relate to the review questions and objectives.	Page 1
**INTRODUCTION**			
Rationale	3	Describe the rationale for the review in the context of what is already known. Explain why the review questions/objectives lend themselves to a scoping review approach.	Page 4
Objectives	4	Provide an explicit statement of the questions and objectives being addressed with reference to their key elements (e.g., population or participants, concepts, and context) or other relevant key elements used to conceptualize the review questions and/or objectives.	Page 4
**METHODS**			
Protocol and registration	5	Indicate whether a review protocol exists; state if and where it can be accessed (e.g., a Web address); and if available, provide registration information, including the registration number.	n/a
Eligibility criteria	6	Specify characteristics of the sources of evidence used as eligibility criteria (e.g., years considered, language, and publication status), and provide a rationale.	Page 4
Information sources*	7	Describe all information sources in the search (e.g., databases with dates of coverage and contact with authors to identify additional sources), as well as the date the most recent search was executed.	Page 4
Search	8	Present the full electronic search strategy for at least 1 database, including any limits used, such that it could be repeated.	Page 4
Selection of sources of evidence†	9	State the process for selecting sources of evidence (i.e., screening and eligibility) included in the scoping review.	Pages 4,5
Data charting process‡	10	Describe the methods of charting data from the included sources of evidence (e.g., calibrated forms or forms that have been tested by the team before their use, and whether data charting was done independently or in duplicate) and any processes for obtaining and confirming data from investigators.	Page 5
Data items	11	List and define all variables for which data were sought and any assumptions and simplifications made.	Click here to enter text.
Critical appraisal of individual sources of evidence§	12	If done, provide a rationale for conducting a critical appraisal of included sources of evidence; describe the methods used and how this information was used in any data synthesis (if appropriate).	Page 5

SECTION	ITEM	PRISMA-ScR CHECKLIST ITEM	REPORTED ON PAGE #
Synthesis of results	13	Describe the methods of handling and summarizing the data that were charted.	Page 5
**RESULTS**			
Selection of sources of evidence	14	Give numbers of sources of evidence screened, assessed for eligibility, and included in the review, with reasons for exclusions at each stage, ideally using a flow diagram.	Page 6
Characteristics of sources of evidence	15	For each source of evidence, present characteristics for which data were charted and provide the citations.	Page 7, Annex 2
Critical appraisal within sources of evidence	16	If done, present data on critical appraisal of included sources of evidence (see item 12).	Page 5, Annex 1
Results of individual sources of evidence	17	For each included source of evidence, present the relevant data that were charted that relate to the review questions and objectives.	Annex 2
Synthesis of results	18	Summarize and/or present the charting results as they relate to the review questions and objectives.	Pages 7-17
**DISCUSSION**			
Summary of evidence	19	Summarize the main results (including an overview of concepts, themes, and types of evidence available), link to the review questions and objectives, and consider the relevance to key groups.	Pages 17-20
Limitations	20	Discuss the limitations of the scoping review process.	Page 20
Conclusions	21	Provide a general interpretation of the results with respect to the review questions and objectives, as well as potential implications and/or next steps.	Pages 20-21
**FUNDING**			
Funding	22	Describe sources of funding for the included sources of evidence, as well as sources of funding for the scoping review. Describe the role of the funders of the scoping review.	Page 25

JBI = Joanna Briggs Institute; PRISMA-ScR = Preferred Reporting Items for Systematic reviews and Meta-Analyses extension for Scoping Reviews.

* Where *sources of evidence* (see second footnote) are compiled from, such as bibliographic databases, social media platforms, and Web sites.

† A more inclusive/heterogeneous term used to account for the different types of evidence or data sources (e.g., quantitative and/or qualitative research, expert opinion, and policy documents) that may be eligible in a scoping review as opposed to only studies. This is not to be confused with *information sources* (see first footnote).

‡ The frameworks by Arksey and O'Malley (6) and Levac and colleagues (7) and the JBI guidance (4, 5) refer to the process of data extraction in a scoping review as data charting.

§ The process of systematically examining research evidence to assess its validity, results, and relevance before using it to inform a decision. This term is used for items 12 and 19 instead of "risk of bias" (which is more applicable to systematic reviews of interventions) to include and acknowledge the various sources of evidence that may be used in a scoping review (e.g., quantitative and/or qualitative research, expert opinion, and policy document).

From: Tricco AC, Lillie E, Zarin W, O'Brien KK, Colquhoun H, Levac D, et al. PRISMA Extension for Scoping Reviews (PRISMAScR): Checklist and Explanation. Ann Intern Med. 2018;169:467–473. doi: 10.7326/M18-0850.
